# Intercellular Genetic Interaction Between *Irf6* and *Twist1* during Craniofacial Development

**DOI:** 10.1038/s41598-017-06310-z

**Published:** 2017-08-02

**Authors:** Walid D. Fakhouri, Kareem Metwalli, Ali Naji, Sarah Bakhiet, Angela Quispe-Salcedo, Larissa Nitschke, Youssef A. Kousa, Brian C. Schutte

**Affiliations:** 1 Center for Craniofacial Research, Department of Diagnostic and Biomedical Sciences, School of Dentistry, University of Texas Health Science Center at Houston, TX, 77054 USA; 2 Department of Pediatrics, Medical School, University of Texas Health Science Center at Houston, TX, 77030 USA; 3 Graduate School of Biomedical Sciences, University of Texas Health Science Center and MD Anderson Cancer Center at Houston, TX, 77030 USA; 4 Microbiology and Molecular Genetics, Michigan State University, East Lansing, MI 48823 USA; 5 Department of Biochemistry and Molecular Biology, Michigan State University, East Lansing, MI 48823 USA; 6 Pediatrics and Human Development, Michigan State University, East Lansing, MI 48823 USA; 7 Department of Basic Science, School of Dentistry, National University of San Marcos (UNMSM), Lima, Peru; 8 Program in Integrative Molecular and Biomedical Sciences, Baylor College of Medicine, Houston, TX 77030 USA; 9 Pediatric Residency Program, Children’s National Health System, Washington, DC 20010 USA

## Abstract

Interferon Regulatory Factor 6 (*IRF6*) and *TWIST1* are transcription factors necessary for craniofacial development. Human genetic studies showed that mutations in *IRF6* lead to cleft lip and palate and mandibular abnormalities. In the mouse, we found that loss of *Irf6* causes craniosynostosis and mandibular hypoplasia. Similarly, mutations in *TWIST1* cause craniosynostosis, mandibular hypoplasia and cleft palate. Based on this phenotypic overlap, we asked if *Irf6* and *Twist1* interact genetically during craniofacial formation. While single heterozygous mice are normal, double heterozygous embryos (*Irf6* ^+*/−*^; *Twist1* ^+*/−*^) can have severe mandibular hypoplasia that leads to agnathia and cleft palate at birth. Analysis of spatiotemporal expression showed that *Irf6* and *Twist1* are found in different cell types. Consistent with the intercellular interaction, we found reduced expression of Endothelin1 (EDN1) in mandible and transcription factors that are critical for mandibular patterning including DLX5, DLX6 and HAND2, were also reduced in mesenchymal cells. Treatment of mandibular explants with exogenous EDN1 peptides partially rescued abnormalities in Meckel’s cartilage. In addition, partial rescue was observed when double heterozygous embryos also carried a null allele of *p53*. Considering that variants in *IRF6* and *TWIST1* contribute to human craniofacial defects, this gene-gene interaction may have implications on craniofacial disorders.

## Introduction

Development of the face is a highly coordinated process that starts after gastrulation with the formation of the frontonasal and pharyngeal arches. The arches are lined by ectoderm superficially and endoderm internally. Cranial neural crest (CNC) cells that may be considered a fourth germ-layer, migrate from the mid- and hindbrain regions into the frontonasal and pharyngeal arches. CNC cells than give rise to many tissues, including bone, cartilage, neuron, ganglia, smooth muscle and odontoblasts^1, 2^. In the mandible, CNC and cranial paraxial mesodermal cells contribute to the formation of Meckel’s cartilage and the tongue^3–6^. Meckel’s cartilage provides structural support and biochemical cues for CNC-derived mesenchyme during mandibular development^4, 7^. Also, signaling from oral epithelium is critical in regulating proliferation and differentiation of CNC^6, 8^. Hence, the development of craniofacial tissues is highly coordinated and interconnected. For example, micrognathia (undersized mandible) can lead to glossoptosis and cleft palate, as seen in patients with Pierre Robin sequence^9, 10^.

Mandibular disorders are common congenital malformations that can occur as part of genetic syndromes or as an isolated form that can lead to a sequence of anomalies associated with tongue, palate and pharynx^11, 12^. The phenotypic expression of mandibular abnormalities ranges from hyperplasia (macrognathia) to hypoplasia (micrognathia) and to a more severe form characterized by total loss of the mandible (agnathia)^10–12^. Micrognathia is the most common mandibular birth defect with a frequency of 1 in 1600 live births^12^. Although jaw disorders drastically affect the quality of life and are associated with high morbidity and mortality in severe cases^10, 11, 13^ little is known about the genetic and environmental risk factors that contribute to their pathology in humans^14–16^. An important transcription factor in for mandibular and palatal development is *TWIST1*, which encodes a basic helix-loop-helix protein that is expressed in neural crest-derived mesenchyme during craniofacial development^17, 18^. Mutations in *TWIST1* cause Saethre Chotzen syndrome (SCS; OMIM#101400), which is characterized by premature fusion of fibrous sutures in the skull and is the most common syndromic form of craniosynostosis^19^. Individuals with SCS can also have an asymmetric, hypoplastic face^19^, approximately 6% have cleft palate and 43% have high-arched palates^20^. However, a biological rationale for the association between craniosynostosis and cleft palate has not been delineated yet^20, 21^. In mice, loss of *Twist1* is embryonic lethal at E11.5^18^. *Twist1* heterozygous pups can have preaxial polydactyly in the hind limbs (30–50%) but do not have craniofacial defects at birth^22^. Conditional knockout of *Twist1* in cranial neural crest cells results in severe mandibular hypoplasia, cleft palate and loss of frontal and maxillary bones^18^. These data show that *Twist1* is critical in tissues derived from the first pharyngeal arch.

Unlike other Interferon Regulatory Factors, *IRF6* is broadly expressed during embryonic development, including the oral epithelium, medial edges of palatal shelves and developing skin^23–25^. Consistent with this, mutations in *IRF6* can lead to cleft lip and palate, as well as skin and limb abnormalities in Van der Woude and Popliteal Ptyergium syndromes (OMIM #119300 and 119500)^26^. A common variant in *IRF6* also contributes 12–18% of the risk associated with isolated cleft lip with/without cleft palate^27, 28^. In mice, embryos that are heterozygous for *Irf6* null allele are grossly normal. Interestingly, homozygous null of *Irf6* leads to multiple skeletal defects involving the xiphoid process, limbs, digits and mandible. However, *Irf6* expression has not been detected in bone tissues suggesting intercellular communication^23, 24^. Currently, it is not clear how *Irf6*, a transcription factor that is primarily expressed in epithelial cells, could perturb the development of adjacent mesenchymal cells in craniofacial and bone development.

Based on the phenotypic overlap in humans and mice, we investigated a potential genetic interaction between *Irf6* and *Twist1* during craniofacial development. We intercrossed *Irf6* and *Twist1* heterozygous mice to obtain pups that are double heterozygous for *Twist1* and *Irf6*. Although individual heterozygous embryos for *Irf6* or *Twist1* show no phenotype at birth, the double heterozygous pups had a severe mandible abnormality and cleft of the secondary palate. We investigated the possibility of a direct genetic interaction because the *IRF6* enhancer element contains five TWIST1 putative binding motifs^29^. We examined this mechanism using cell culture of human keratinocytes and embryonic epithelial cells and found that TWIST1 binds to *IRF6* enhancer element and negatively suppresses its expression. We also considered intercellular communication because *Irf6* is not expressed in bony tissues that are affected in *Irf6* knockout mice. As a result, we reanalyzed the skeletal phenotype in *Irf6* null embryos and investigated expression of select genes in *Irf6* null and compound alleles for *Irf6* null and *p53* heterozygous mice (*Irf6* ^−/−^; *p53* ^+*/−*^). We then conducted *in vivo* and *ex vivo studies* to further characterize the phenotype and signaling pathway in murine embryos to better understand the nature of this novel interaction and to rescue the phenotype genetically and pharmacologically.

## Results

### *Irf6* and *Twist1* interact genetically during mandibular development

**Figure 1:**
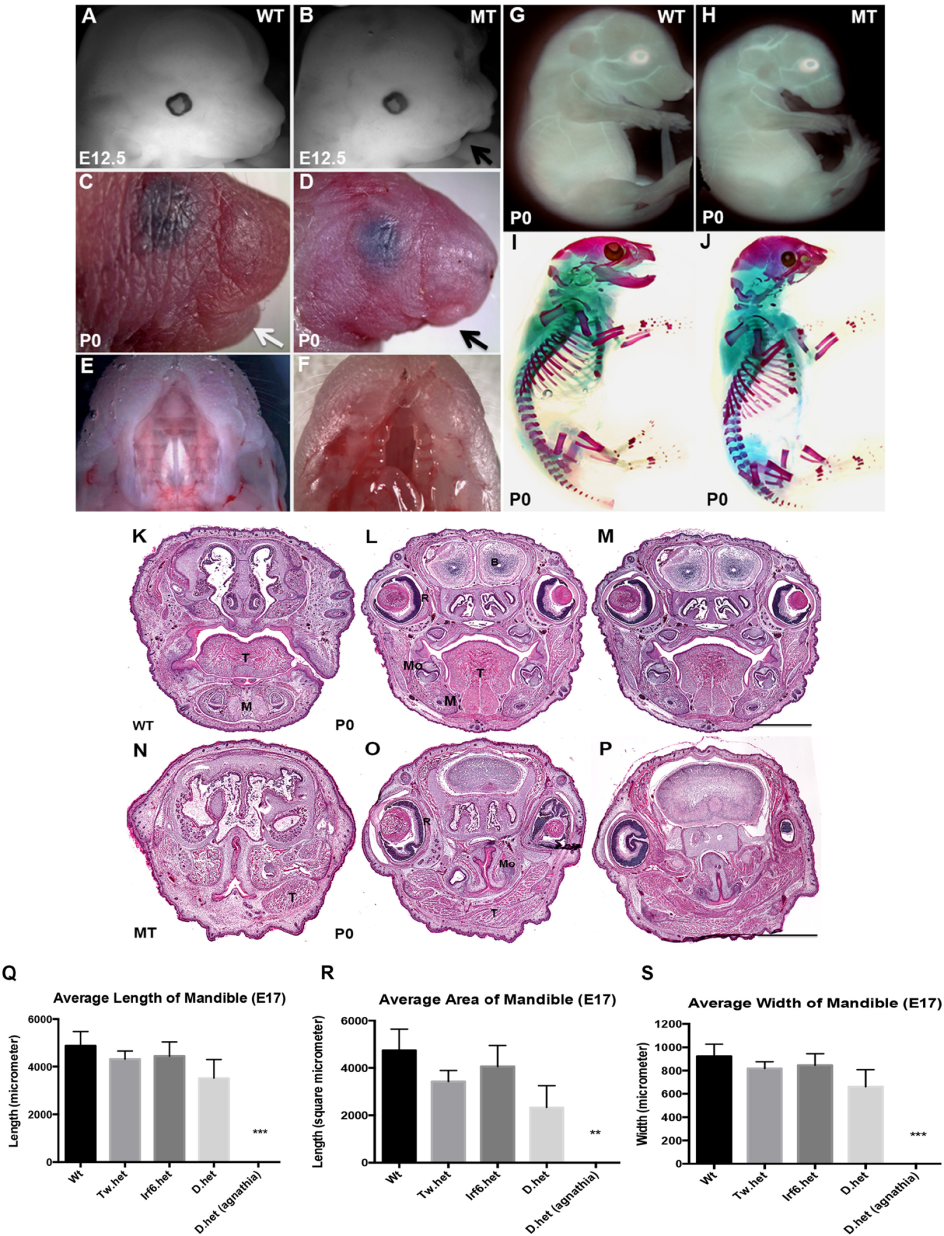
Craniofacial abnormalities in *Irf6* and *Twist1* double heterozygotes murine animals and morphometric measurements of embryonic mandible. Compared to wild type murine embryo (**A**), littermate mutant embryo of double het (**B**) has a short mandibular prominence start at E12.5. At P0, mutant pup has lower jaw abnormalities (**C** versus **D**), and complete cleft of the secondary palate (**E** versus **F**). Phase contrast images show mutant embryo with absent lower jaw and low-set ear (**H**) but grossly normal body (**G** versus **H**). Skeletal preparations show that the mutant pup is missing a mandible and has abnormalities in inner ear bones (**I** versus **J**). Hematoxylin and eosin staining of coronal sections of heads from wild type (**K**–**M**) and double heterozygous affected murine pups (**N**–**P**), from anterior (**K**,**N**), middle (**L**,**O**) to posterior (**M**,**P**). Relative to wild type littermate (WT), mutant (MT) has cleft palate, malformed tongue, detached retina and holoprosencephaly (**O**,**P**). Tongue (T), mandible (**M**), molar tooth germ (Mo), retina (R), brain (B). Scale bars = 1000 μm. Figures (**Q**,**R** and **S**) are average length, width and area of murine mandibles of different genotypes at E17.5. A total of 28 craniofacial skeletons of murine embryos were used to calculate the averages. The black bars represent standard error for each genotype. Average mandibular lengths of murine embryos of all different genotypes (**Q**). Average mandibular widths of murine embryos of all different genotypes (**R**). Average mandibular area of murine embryos of all different genotypes (**Q**). The average area of *Irf6*, *Twist1* double hets vs. wild type is smaller relative to mean area of wild types, but the difference is not statistically significant using the multi-variants ANOVA test. Wild type (WT), Twist1 heterozygous (Tw.het), Irf6 heterozygous (Irf6.het) and double heterozygous (D.het).

Although single heterozygous (het) mice for either *Irf6* or *Twist1* have grossly normal craniofacial development, pups with a single mutant allele of *Irf6* and *Twist1* (*Irf6*; *Twist1* double hets) can develop severe craniofacial defects, including low-set ears, mandibular abnormalities and cleft palate (Fig. 1A–J). To track this dysmorphology during development, we examined double heterozygous mice for *Irf6* and *Twist1* at different developmental time points. We detected hypoplasia of mandibular prominences as early as E12.5 (Fig. 1A,B and Supplementary Fig. S1). The most severely affected embryos developed agnathia (Fig. 1C,D), U-shape clefting of the secondary palate (Fig. 1E,F) and died shortly after birth. Phase-contrast imaging showed that *Irf6*, *Twist1* double hets were otherwise grossly normal except for the lower jaw (Fig. 1G,H). The skeleton of *Irf6*, *Twist1* double hets confirmed loss of the mandible and showed the low-set ears and abnormalities of inner ear bones (Fig. 1H,J) compared to wild type control (Fig. 1G,I and Supplementary Fig. S2). Despite delay in limb development at E12.5 (Supplementary Fig. S1), the skeletal bone and cartilage did not show a permanent defect (Fig. 1J and Supplementary Fig. S2). Compared to wild type littermate (Fig. 1K,L,M), H&E staining of agnathic mutant pup revealed other abnormalities including a malformed tongue, detached retina and incomplete penetrance of holoprosencephaly in a couple of embryos (Fig. 1N,O,P). We did not detect a difference in the rate of preaxial polydactyly between *Twist1* heterozygous embryos and *Irf6*, *Twist1* double hets (data not shown). From 81 litters of this mating, we obtained 619 embryos and newborn pups, including 61 resorbed embryos. Genotyping showed that 107 embryos and pups were double heterozygotes for *Irf6* ^+*/−*^ *;Twist1* ^+*/−*^, a number that is fewer than expected (p < 0.03), suggesting a prenatal or perinatal lethality. From the 107 double heterozygotes for *Irf6* ^+*/−*^ *;Twist1* ^+*/−*^, 62 embryos and pups were mutant (57.9%).

### Gene dosage effect on length, width and area of mandible

In order to elucidate whether the grossly unaffected double heterozygous embryos have subtle mandibular defects, we measured the length, width and area of mandibles from 28 embryos obtained at E17.5. The embryos were sorted into the following genotypes: wild type (*Irf6* ^+/+^; *Twist1* ^+/+^, n = 7), *Irf6* single heterozygous (*Irf6* ^+*/−*^; *Twist1* ^+/+^, n = 8), *Twist1* single heterozygous (*Irf6* ^+/+^; *Twist1* ^+*/−*^, n = 8), and double heterozygotes (*Irf6* ^+*/−*^; *Twist1* ^+*/−*^, n = 5). Eight agnathic embryos were processed for skeletal preparation but the data were not included in the mandibular analysis due to complete lack of mandible. Using the averages of the lengths, widths, and areas of the mandibles, we detected a quantitative phenotypic difference among the different genotypes and identified a trend, with double heterozygotes generally having the smallest mandibles (Fig. 1Q,R,S). Measurements of area presented the greatest differences between all four genotypes (Fig. 1Q,R,S). The multi-variants ANOVA test for the four genotypes did not show significant difference among the mean values except for the agnathia double heterozygous embryos. However, only paired t-test analysis indicated that the mandibular area of *Irf6*, *Twist1* double hets is smaller relative to mean area of wild types with a p-value of 0.0169. Representative images of mandibular landmarks used in the measurements and craniofacial skeleton of each genotype are depicted in Supplementary Figs S3 and S4.

### TWIST1 suppresses *IRF6* expression ***in vitro*** by binding to its enhancer element

**Figure 2:**
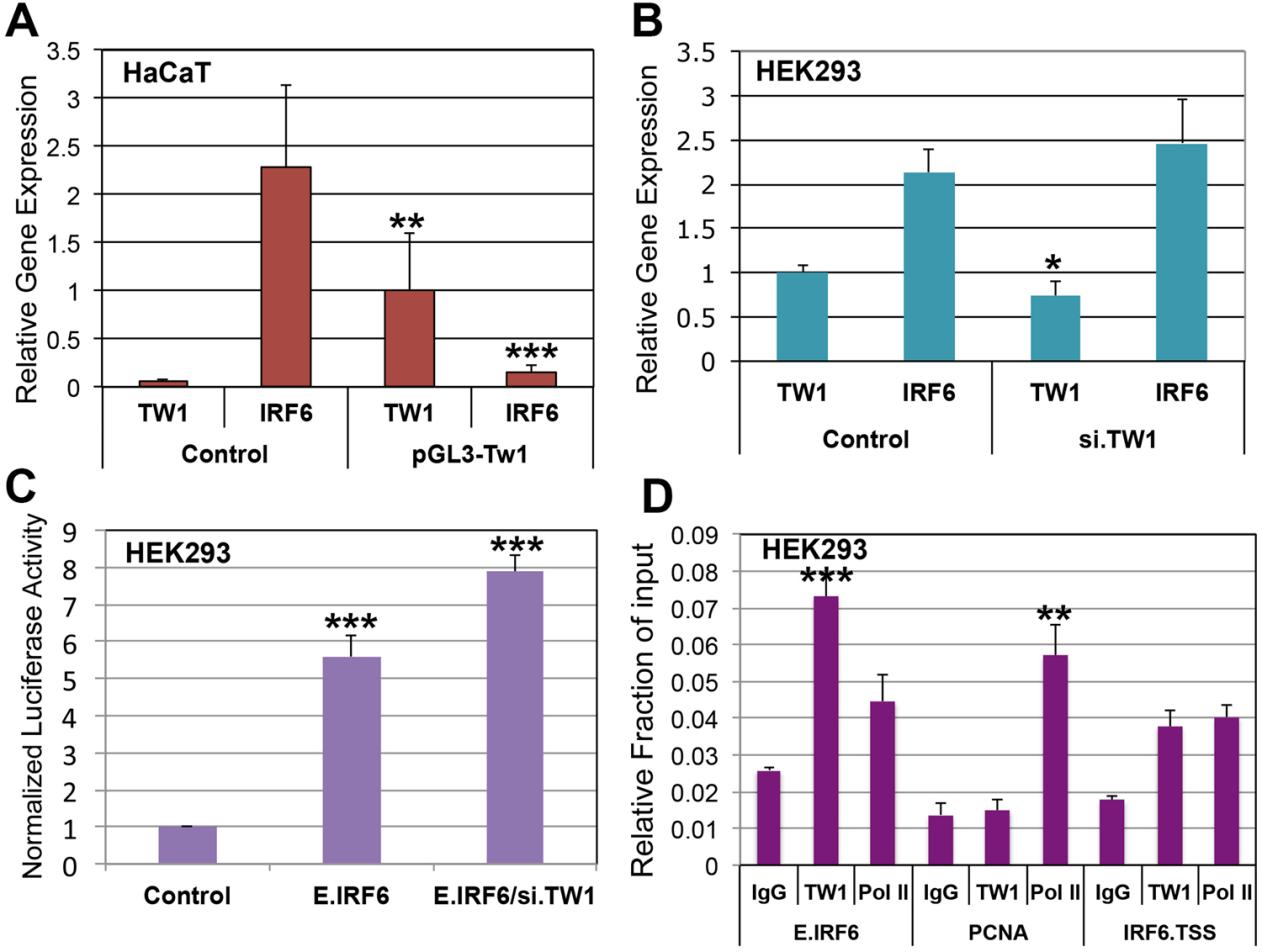
TWIST1 suppresses *IRF6* expression *in vitro* via its enhancer element. RTqPCR data shows that TWIST1 is not expressed in HaCaT keratinocytes, while IRF6 is highly expressed in these cells (**A**). Exogenous overexpression of TWIST1 using pGL3 expression vector remarkably reduced IRF6 expression by 12-fold 48 h post-transfection (**A**). In human embryonic kidney cells (HEK293), TWIST1 is moderately expressed compared to IRF6 that is expressed 2-fold more (**B**). Knockdown of TWIST1 expression with siRNA slightly increased the expression level of IRF6 in HEK293 cells (**B**). However, knockdown of TWIST1 expression significantly increased luciferase activity of a luciferase gene that is under direct control of IRF6 enhancer element (**C**). ChIP-seq data showed that TWIST1 binds to IRF6 enhancer element accompanied by enrichment of RNA PolII protein at the enhancer and promoter element of IRF6 gene, but the enrichment of PolII at the enhancer and promoter regions of IRF6 may indicate an active transcriptional state. However, the amount of PolII at the IRF6 enhancer and promoter compared to IgG was not significant and the enrichment was moderate compared to PolII at PCNA promoter (**D**).

The *IRF6* enhancer element has 5 putative E-box binding sites for TWIST1^29^. We tested whether TWIST1 could directly regulate *IRF6* expression at this enhancer element (MCS9.7). *In vitro* data showed that *TWIST1* is not expressed in human HaCaT keratinocytes, while *IRF6* is highly expressed in these cells (Fig. 2A). However, exogenous overexpression of *TWIST1* in HaCaT significantly reduced *IRF6* expression by more than 12-fold compared to untransfected control cells (Fig. 2A). To determine the effect of loss of function on *IRF6* expression, we used a HEK293 cell line where *TWIST1* basal expression can easily be detected together with *IRF6*. Knockdown of *TWIST1* expression by siRNA increased, but not significantly, *IRF6* mRNA level compared to control cells (Fig. 2B). However, the expression of the luciferase reporter gene driven by the *IRF6* enhancer element was significantly increased upon knockdown of *TWIST1* expression (Fig. 2C). To determine if the effect is direct, ChIP-seq assay was performed to detect the binding of Twist1 to the *Irf6* enhancer element. The ChIP-seq data showed that TWIST1 significantly binds to *IRF6* enhancer suggesting a direct regulation of *IRF6* expression in human HEK293 cell culture (Fig. 2D). Furthermore, enrichment of PolII at the enhancer and promoter regions of IRF6-transcription start site (TSS) may indicate an active transcriptional state of *IRF6* gene. However, the level of PolII at the IRF6 enhancer and promoter compared to IgG was not significant and the enrichment was moderate compared to PolII at PCNA promoter that was used as an internal control for the efficiency of chromatin immunoprecipitation with TWIST1 and PolII antibodies (Fig. 2D).

### IRF6 and TWIST1 are mainly expressed in different cell types ***in vivo***

**Figure 3:**
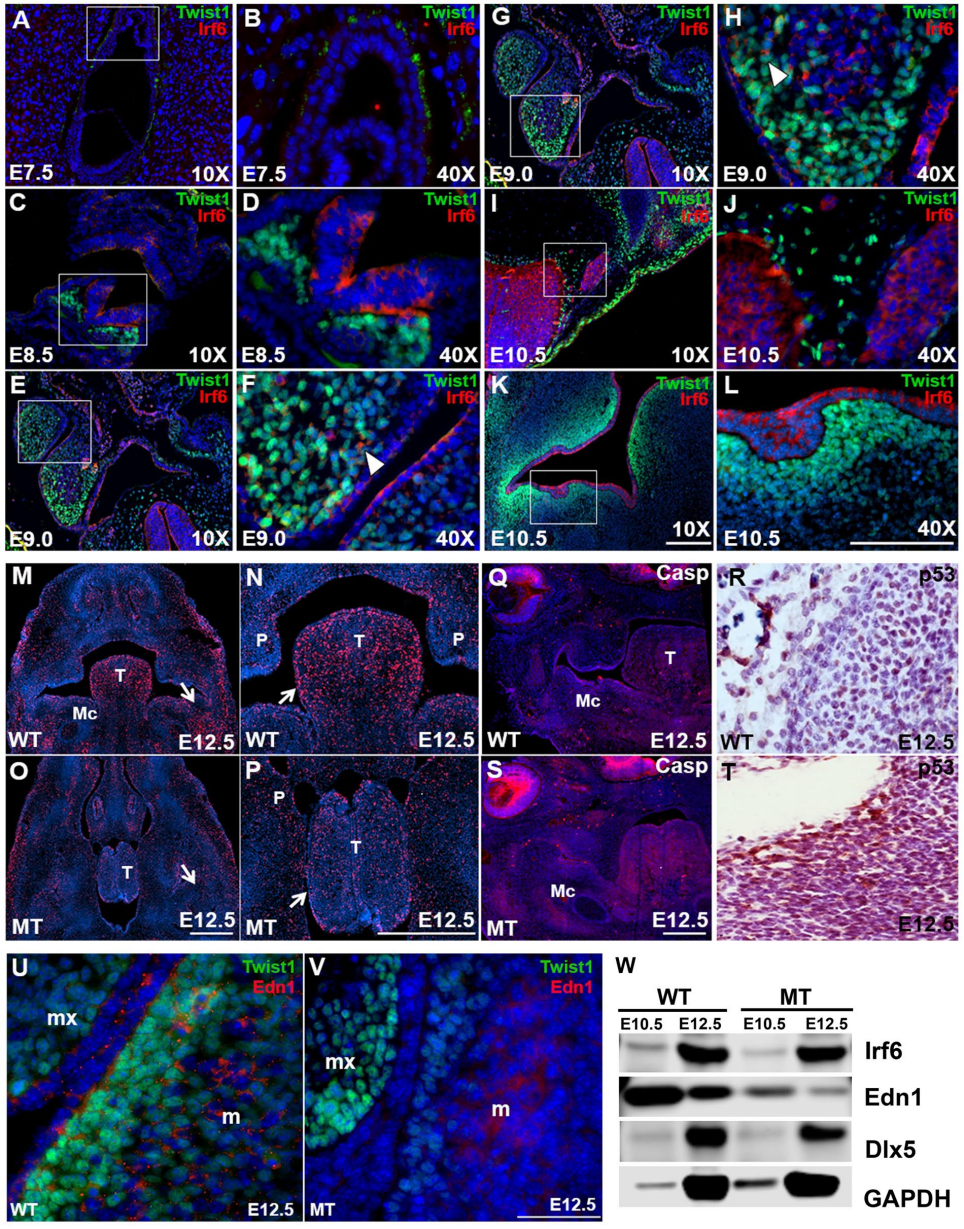
Immunostaining for detection of IRF6 and TWIST1 expression and immunoblot for total protein analysis. IRF6 and TWIST1 are not expressed at E7.5 (**A**,**B**), while their expression is detected in neuroectoderm for IRF6 and in mesoderm for TWIST1 at E8.5 (**C**,**D**). IRF6 expression persists in neuronal- and non-neuronal ectoderm, while TWIST1 expression is detected in CNC cells at E9.0 (**E**,**F**). Cytosolic IRF6 and nuclear TWIST1 are co-localized to subset of CNC cells in first and second pharyngeal arches at E9.0 (White arrowheads, **F**,**H**). Subsequently, IRF6 is expressed in neural tube (**I**,**J**) and oral epithelium (**K**,**L**), while TWIST1 is expressed in migratory CNC cells (**I**,**J**) and mesenchymal cells (**K**,**L**) at E10.5. For assessment of cell proliferation and apoptosis, immunofluorescent images show the level of cell proliferation by highlighting the cells undergoing cell division in red in wild type embryos (**M**,**N**) compared to double heterozygous mutant for *Irf6* and *Twist1* at E12.5 (**O**,**P**). Relative to wild type (**Q**), immunostaining of activated caspase 3 indicates a higher level of cell apoptosis in the mutant at E12.5 (**S**). Stronger signal is observed in retina, tongue and mandible. Immunohistochemical staining show the level of P53 in wild type mandibular tissues (**R**) compared to mutant samples (**T**). For expression of EDN1 and TWIST1, EDN1 is secreted from epithelial and mesodermal cells of the mandible (m) but not in the maxilla (mx) (**U**), while TWIST1 is expressed in the adjacent mesenchymal cells in mandible and maxilla. Relative to wild type (**U**), EDN1 and TWIST1 are significantly reduced in epithelium and CNC cells in mutant samples, respectively, but the expression of TWIST1 in maxilla is not affected (**V**). Scale bars = 100 μm. Immunoblot was performed to validate the IF data of EDN1 at quantitative level. EDN1 proteins were remarkably reduced in mutant mandibular processes compared to wild type littermates at both embryonic stages E10.5 and E12.5 (**W**). Slight reduction in IRF6 expression and DLX5 was noticed at E10.5 and E12.5, respectively (**W**). GAPDH was used as internal loading control and full-length gels are included in supplementary.

We analyzed the spatiotemporal expression pattern of both IRF6 and TWIST1 in wild type embryos to determine the mechanism of interaction during embryonic development. Analysis of *Irf6*, *Twist1* double hets showed dysmorphology as early as E12.5. As a result, we examined expression of *Irf6* and *Twist1* from E7.5 (gastrulation) to E14.5. IRF6 and TWIST1 were not expressed at E7.5 (Fig. 3A,B). At E8.5, IRF6 is expressed in the neuroectoderm and TWIST1 in the adjacent mesodermal cells (Fig. 3C,D). Subsequently, expression of IRF6 was found in neural and non-neural ectoderm, while TWIST1 expression was found in migratory CNC cells (Fig. 3E–J). Interestingly, we observed co-localization of IRF6 and TWIST1 in a subset of CNC migratory cells in both the first and second pharyngeal arches at E9.0 (Fig. 3F,H). Interesting, Irf6 is highly expressed in neural tube, while CNC cells that are transitioned from neural tube boarder express high level of Twist1 during migration (Fig. 3I,J). At E10.5 in the oral cavity, IRF6 was found in oral epithelium while TWIST1 was found in underlying CNC-derived mesenchymal cells that give rise to the cartilage and bone of the jaw (Fig. 3K,L). The expression pattern of IRF6 in oral epithelium and TWIST1 in mandibular CNC-derived mesenchyme persisted for the reminder of embryonic development and into the neonatal period (Supplementary Fig. S5). At least 5 independent biological embryos for each genotype were analyzed for the spatiotemporal expression of IRF6 and TWIST1.

### Reduced proliferation and increased cell death in *Irf6*, *Twist1* double hets

Considering a smaller mandible beginning at E12.5, we considered the role of cell death and proliferation in *Irf6*, *Twist1* double hets. Using BrdU as a marker for proliferation, we observed numerous BrdU-positive cells in the tongue and around Meckel’s cartilage in wild type embryos at E12.5 (Fig. 3M,N). In contrast, fewer BrdU-positive cells were detected in *Irf6*, *Twist1* double hets at an analogous plane of section (Fig. 3O,P). These data suggest that reduced cellular proliferation maybe contributing to hypoplastic mandibles. We also considered the role of cell death and used activated-Caspase 3 (a-CASP3) as a marker for apoptosis. Compared to wild type littermates of other dams (Fig. 3Q), the a-CASP3 signal was greatly increased in the retina, tongue and mandibular tissues of *Irf6*, *Twist1* double hets (Fig. 3S). With increased cell death according to a-CASP3 immunostaining, we considered the role of p53 in this pathway. Consistent with this rationale, we found a stronger P53 signal in *Irf6*, *Twist1* double het embryos (Fig. 3T) compared to wild type littermates (Fig. 3R). Importantly, the P53 signal positively correlated with cell death at E12.5. In contrast, dual staining showed Twist1 to be negatively correlated with cell apoptotic markers P53 and BAX in oral tissues (Supplementary Fig. S6). Altogether, our data shows a relative reduction in TWIST1 and cell proliferation with a corresponding increase in the apoptotic markers A-CASP3, P53 and BAX in mandibular and tongue tissues.

### Disruption of Endothelin1 signaling in *Irf6*, *Twist1* double hets

With expression of IRF6 in mandibular epithelium and TWIST1 in CNC-derived mesenchyme starting at E9.5 and delayed mandibular development at E12.5, we predicted an intercellular interaction. During mandibular development, multiple signaling pathways are active between the epithelium and cranial neural crest cells, including EDN1. We performed dual immunostaining of TWIST1 and EDN1 at E12.5. In mandibular and maxillary CNC-derived mesenchyme, we found TWIST1 expression in the nucleus of CNC-derived mesenchymal cells (Fig. 3U). We also detected punctuate EDN1 expression in apical oral epithelium and in mesoderm of the mandible but not in the maxilla (Fig. 3U). Punctuate EDN1 expression at the periphery of CNC cells adjacent to oral epithelium suggested extrusion of a ligand into the intercellular matrix and binding to its cognate receptor (Fig. 3U). TWIST1 and EDN1 expression were both reduced in mandibular CNC cells of mutant embryos (Fig. 3V). We also performed immunoblot analysis to validate the IF staining data at a quantitative level. EDN1 protein was remarkably reduced in mandibular processes of affected double hets at E10.5 and E12.5 compared to wild type littermates (Fig. 3W). However, DLX5 protein level was slightly reduced in affected double hets at E12.5 compared to wild type (Fig. 3W). Full-length western blots and band intensities of each protein were quantified and normalization to housekeeping protein GAPDH to determine fold decrease in protein levels (Supplementary Fig. S7).

### Mandibular hypoplasia, craniosynostosis and reduced *Twist1* expression in *Irf6* null mice

**Figure 4:**
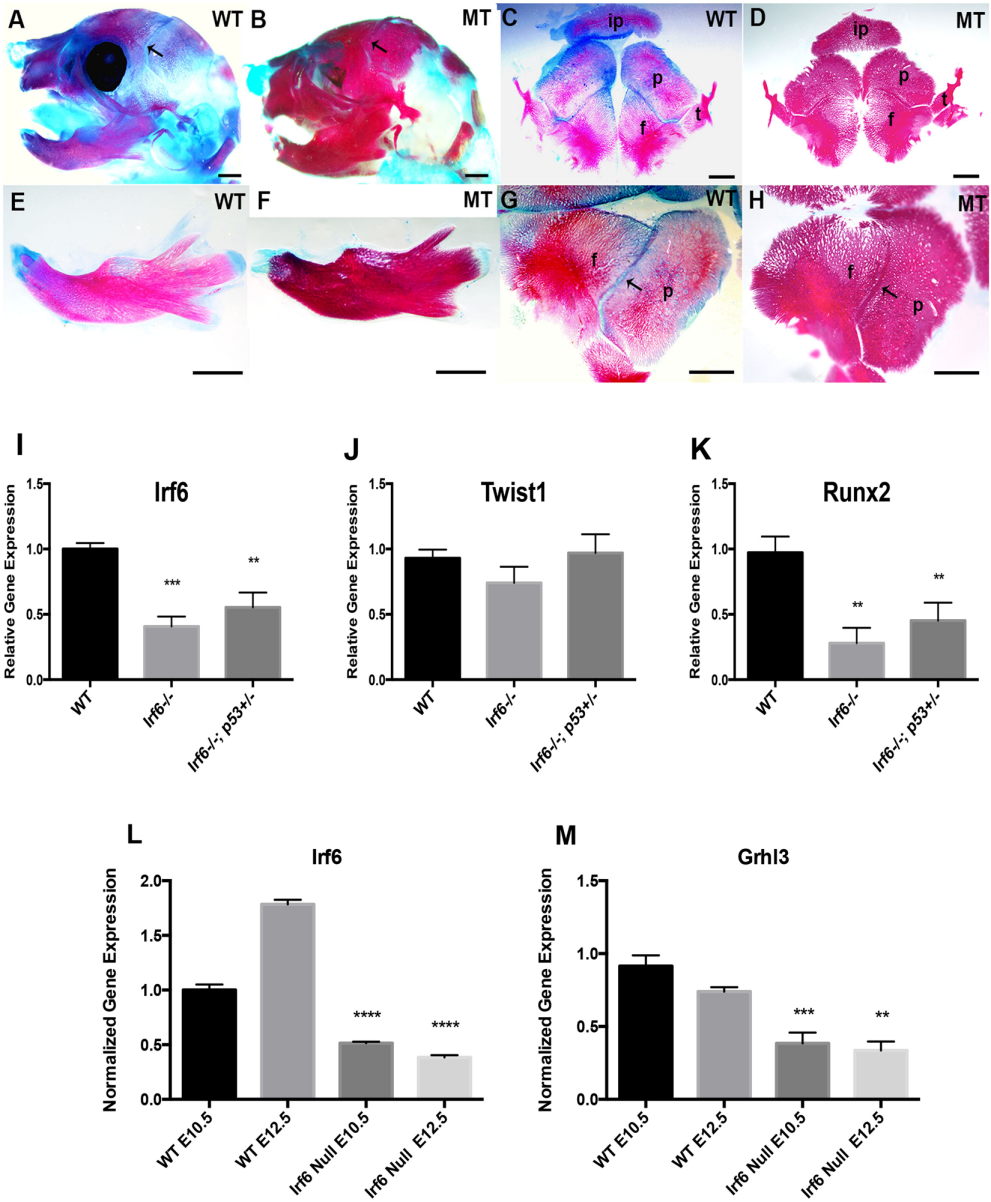
Craniofacial skeletal abnormalities in *Irf6* null pups and quantitative expression of selected genes. (**A**–**H**) Bone (red) and cartilage (blue) of head and mandible of P0 pups. Relative to wild type littermates (**A**,**C**,**E**,**G**), mutant pups (**B**,**D**,**F**,**H**) have less cartilage (**B**,**F**), and the mandible is missing a distal bone where the incisor is normally formed (**E** versus **F**). The mandibular processes are shorter (**F**). The coronal sutures of the skull of Irf6 null pups are fused (arrows) (**D**,**H**) compared to wild type littermate (**C**,**G**). Scale bars: 1000 μm. Expression of *Irf6*, *Tw1* and *Runx2* is reduced in *Irf6* null embryos at E14.5 compared to wild type littermate (**I**–**K**). Similarly, the expression of *Irf6* and *Runx2* genes is reduced to a lesser extent in compound alleles for *Irf6* null and *p53* heterozygous embryos at E14.5, except for *Tw1* where the expression is slightly increased (**J**). The relative gene expression is the average of two independent experiments with four technical replicates for each genotype. The asterisks represent the level of statistical significance of the average of gene expression compared to wild type embryos. The data are the average of two independent experiments. Similarly, the expression of *Irf6* and *Grhl3* is significantly reduced in *Irf6* null embryos at both E10.5 and E12.5 (**L**,**M**).

Our previous studies suggested skeletal abnormalities in *Irf6* null embryos. To better characterize possible craniofacial defects, we generated skeletal preparations of wild type and *Irf6* null embryos and pups. The mandible and the skull were separated to better visualize the mandibular processes, condyle cartilage, incisor and the skull sutures (Fig. 4A–H). Compared to skeletal preparation of wild type littermate pups (Fig. 4A,C,E,G), we found that loss of *Irf6* causes mandibular hypoplasia (Fig. 4B,D) and craniosynostosis (Fig. 4F,H). Shortened mandibles in *Irf6* null embryos were missing the tip of the incisor, associated with bone loss at the distal segment and absence of condyle cartilage (Fig. 4B,D). Analysis of skull bones showed a fusion of the coronal suture between petrous part of the temporal bone and the frontal bone (Fig. 4F,H) compared to wild type littermates (Fig. 4E,G). Analysis of critical genes in mandibular development from *Irf6* null embryos showed that *Irf6*, *Twist1* and *Runx2* expression were reduced at E14.5 (Fig. 4I–K). Interestingly, the reduced expression level of *Irf6*, *Twist1* and *Runx2* observed in *Irf6* null embryos was relatively increased when a *p53* heterozygous allele was introduced in *Irf6* null embryos (*Irf6* ^−/−^ *; p53* ^*−/*+^) (Fig. 4I–K). Similarly, the expression of *Grhl3* was significantly reduced in *Irf6* null embryos at E10.5 and E12.5, in which Irf6 expression was used as internal control and showed significant reduction (Fig. 4L,M).

### Disruption in the expression of Endothelin1 downstream targets

**Figure 5:**
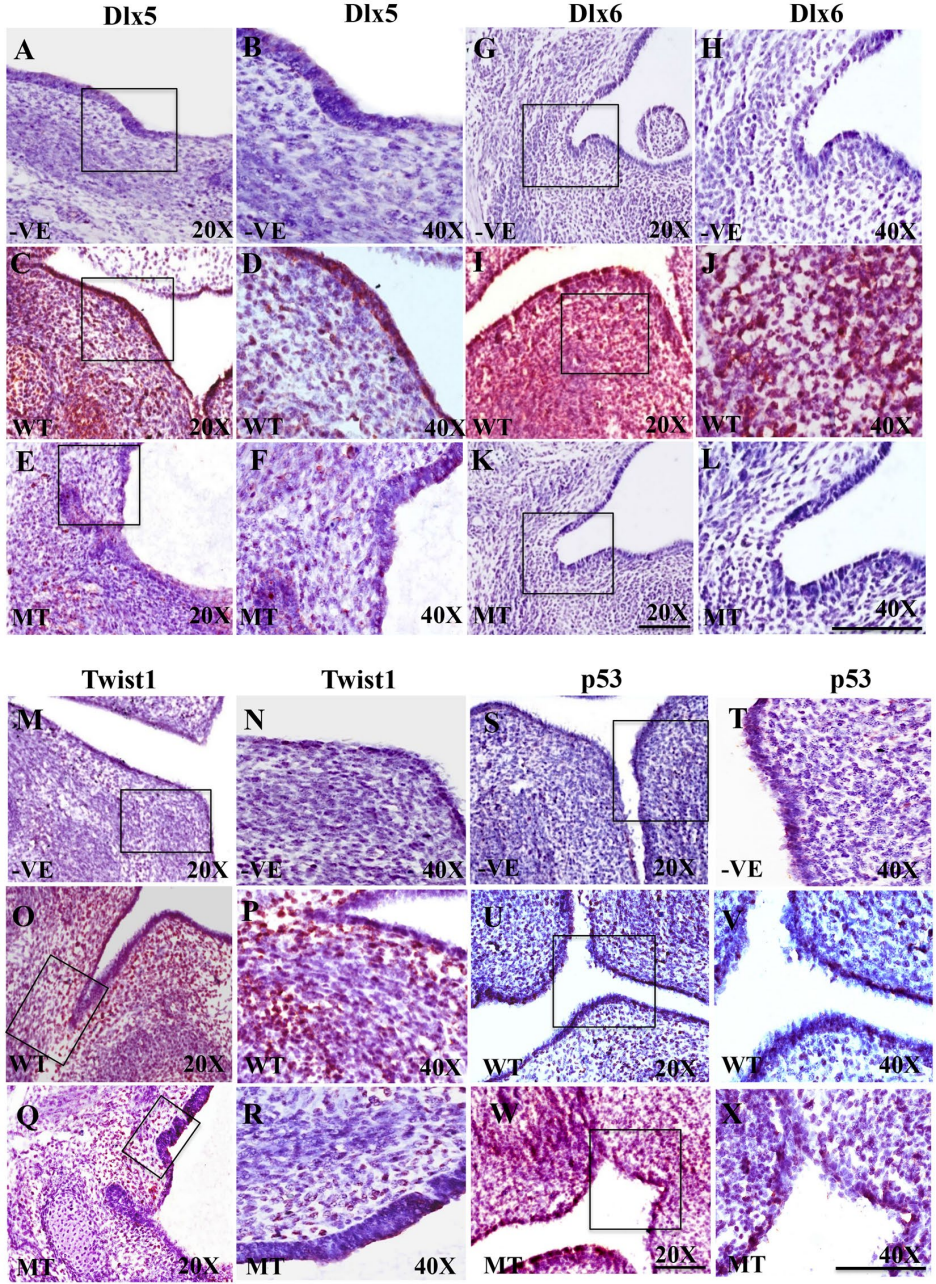
Protein expression of DLX5, DLX6, TWIST1 and P53 in mandibular tissues. The counter staining color used in the immunohistochemistry is purple for nuclei and protein expression is in red. Staining without primary antibodies was used as negative control (**A**,**B**,**G**,**H**,**M**,**N**,**S**,**T**). Expression intensity of DLX5 (**C**,**D**), DLX6 (**I**,**J**) and TWIST1 (**O**,**P**) in wild type tissues is reduced compared to double heterozygous mutant embryos for DLX5 (**E**,**F**), DLX6 (**K**,**L**), and TWIST1 (**Q**,**R**) in mandibular processes at E12.5. In contrary, expression of P53 is low in wild type (**U**,**V**), but increased in mutant (**W**,**X**) at E12.5. Scale bars = 20 μm.

To determine the effect on other downstream targets in EDN1 signaling pathway, we looked at the expression of DLX5 and DLX6 canonical downstream targets, using immunohistochemistry (IHC) staining. Negative controls without the primary antibodies were used to determine signaling specificity and signal to noise ratio (Fig. 5A,B,G,H,M,N,S,T). Imaging of wild type embryos showed nuclear localization of DLX5 and DLX6 in both CNC-derived mesenchymal and epithelial cells, consistent with known roles in transcription (Fig. 5C,D,I,J). In contrast, we qualitatively found reduced expression of DLX5 (Fig. 5E,F) and DLX6 (Fig. 5K,L) in mutant littermate embryos. Since we observed increased apoptosis in the mandible of *Irf6*, *Twist1* double heterozygotes, we determined again the expression level of TWIST1 and P53, both in wild type and mutant embryos at E12.5 using IHC, which produces less noise compared to immunofluorescence (Supplementary Fig. S6). While a robust signal of TWIST1 was observed in wild type CNC-derived mesenchymal cells (Fig. 5O,P), TWIST1 expression appeared reduced in mandibular cells of affected double heterozygous tissues (Fig. 5Q,R). In contrast, the level of p53 was near background in wild type littermates (Fig. 5U,V), but appeared stronger in mutant embryos (Fig. 5W,X). The IHC staining was repeated in other biological samples including the expression of HAND2 and showed a similar trend to DLX5 and DLX6 (Supplementary Fig. S8A–L).

### Reduction of cell apoptosis rescues the mandibular defects

Previous studies demonstrated that p53 directly interacts with TWIST1 to inhibit transcriptional activity in cancer cells^30^. Importantly, p53 is a potent cell cycle regulatory and pro-apoptotic factor that suppresses TWIST1 and inhibits proliferation by promoting apoptosis. These results were consistent with the IHC (Fig. 5M–X) and dual immunofluorescent staining for TWIST1 and p53 (Supplementary Fig. S6A,B). Since *Twist1* and *p53* function antagonistically, we tested whether a null allele of p53 would rescue agnathia in the *Irf6, Twist1* double heterozygotes. We observed agnathia in 55% (21/38) of double heterozygous embryos but only 3% (1/35) in the *p53*, *Irf6*, *Twist1* triple heterozygous embryos (Fisher’s exact test p-value = 1 × 10^−4^). Interestingly, the reduced expression level of *Irf6*, *Twist1* and *Runx2* observed in *Irf6* null embryos was partially rescued when a *p53* heterozygous allele was introduced in *Irf6* null embryos (*Irf6* ^−/−^ *; p53* ^*−/*+^) (Fig. 4I–K).

### Meckel’s cartilage hypoplasia in *Irf6*, *Twist1* double hets is rescued by ***ex-vivo*** treatment with EDN1

**Figure 6:**
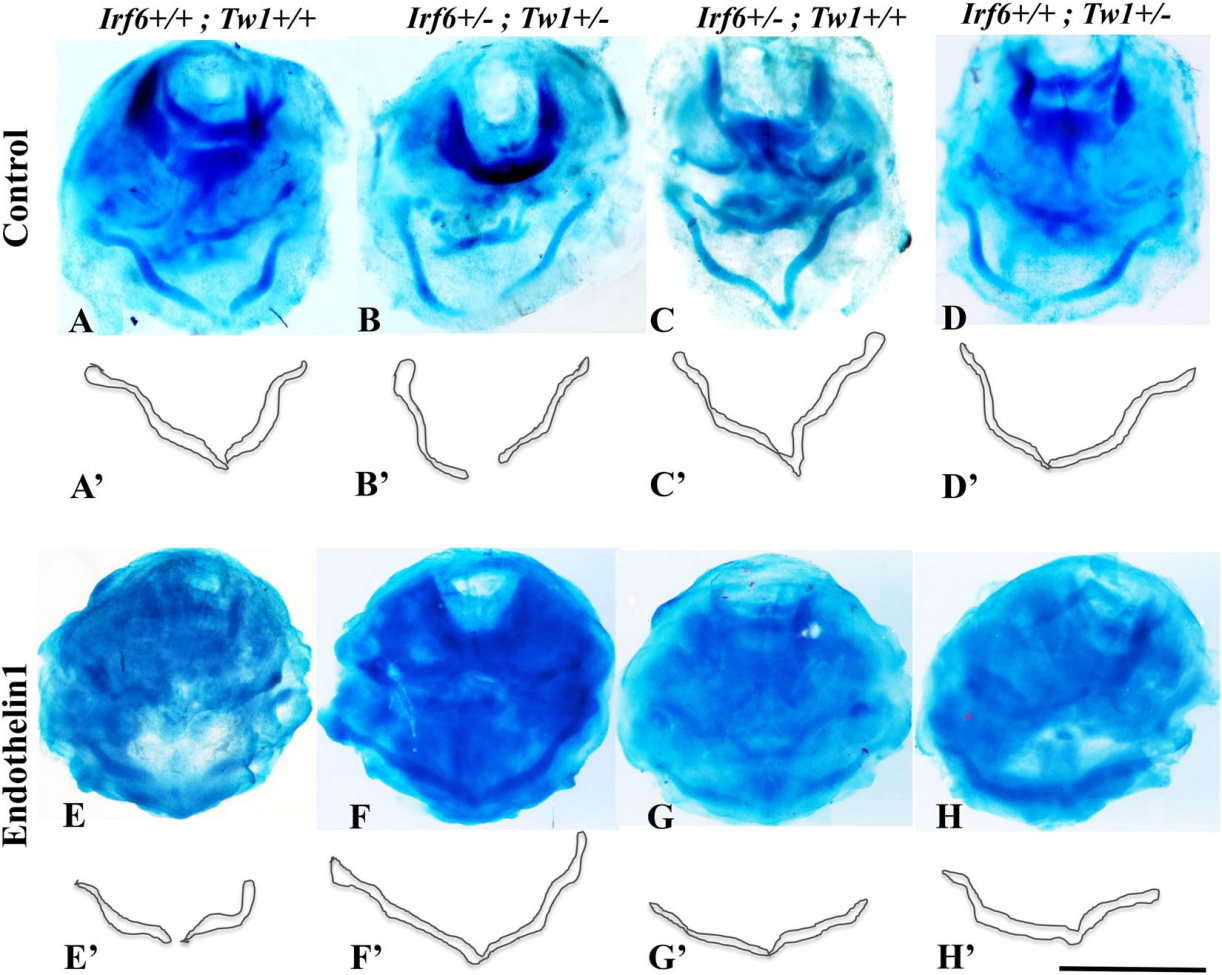
*Ex-vivo* mandibular organ for the rescue experiment with Endothelin1. Top row shows the Meckel’s cartilage of mandibular explants that were cultured in minimal medium without any treatment for all different genotypes (**A**–**D**). Schematic drawing for the Meckel’s cartilage is depicted in (**A**’–**D’**) images for illustrative purposes. The third row shows the Meckel’s cartilage of explants of all different genotypes treated with exogenous Edn1 peptides (**E**–**H**). Similarly, images (**E’**–**H’**) are schematic drawing of the Meckel’s cartilage. Scale bar = 1.5 mm.

Our data suggested that reduced EDN1 expression resulted in abnormal downstream signaling. We used an *ex-vivo* assay to test if exogenous EDN1 peptides could rescue double het mandibular explants. The wild type, double het explants and single hets were grown normally for 4 days (Fig. 6A,B,C,D). We performed rescue experiment with exogenous EDN1 peptide at early stages of mandibular development when EDN1 expression is still important. Treatment of these samples with exogenous EDN1 partially rescued Meckel’s cartilage development in *Irf6*, *Twist1* double hets suggesting partial rescue of mandibular hypoplasia at early gestational stages (Fig. 6E–H). EDN1 treatment also enhanced the size of Meckel’s cartilage in mandibular explants compared to the no treatment group (Supplementary Fig. S9A). Representative results from three different experiments with very similar results are shown in Fig. 6.

## Discussion

We identified a genetic interaction between *Irf6* and *Twist1*, two genes that are essential for craniofacial development. While single heterozygous mice for *Irf6* and *Twist1* are grossly normal, our data suggests a genetic interaction in *Irf6, Twist1* compound heterozygous mice that can lead to severe micrognathia and cleft palate. This interaction is important for mandibular bone development without having a global defect on tissues derived from neural crest cells because the abnormality appeared to be limited to lower jaw and to some extent to the inner ear bones. However, 52% of the newborn double heterozygous pups showed no gross abnormalities indicating incomplete penetrance of the phenotype. Our data resembles the phenotype of Smad2 heterozygous mice where 20% of the newborn pups lack mandibles and eyes, suggesting a dosage dependent effect^31^.

Furthermore, our data suggests that interaction between IRF6 and TWIST1 is primarily intercellular, with a possible contribution from a direct cell autonomous mechanism as well. First, *Irf6* null pups display mandibular hypoplasia and craniosynostosis although IRF6 is not expressed in early mandible or in skull bones, suggesting a non-cell-autonomous effect. Furthermore, IRF6 blocks proliferation and induces terminal differentiation^23^ and one would expect a direct affect on the mandible to lead to larger mandible. Second, immunostaining data showed that *Irf6* and *Twist1* are primarily expressed in adjacent cell types and only transiently co-expressed in the same cell type. Furthermore, *in vitro* data suggests a repressive mechanism on IRF6 by TWIST1. Therefore, loss of a Twist1 allele would therefore potentially lead to more IRF6 expression, which is not consistent with the phenotype observed. Further, we found no evidence for exocrine transport of IRF6 and TWIST1 between the cell types examined. Fourth, we observed a reduction in the expression of EDN1, a ligand that signals from the epithelium and mesoderm to cranial neural crest cells and has been shown to be reduced in double heterozygous mutant embryos. Importantly, reduction of EDN1 was associated with a reduction of DLX5, DLX6 and HAND2, critical factors for mandibular patterning and downstream targets of EDN1 in mesenchymal cells (Figs 3 and 5 and Supplementary Fig. S8). Finally, the addition of EDN1 peptide partially rescued the mandibular development at E10.5-E11.5 in the organ culture model. For these reasons, we favor a model whereby the majority of the affect on the mandible from IRF6 and TWIST1 interaction stems from an intercellular pathway.

During mandibular development, multiple signaling pathways are active between the epithelium and cranial neural crest cells, including Wnt, Tgfβ, Shh, Bmp, Fgf and Edn1^6, 32–35^. We chose to focus on the Endothelin1 pathway based on a number of important clues. First, IRF6 regulates the expression of EDN1 in primary human keratinocytes^36^. Second, Endothelin1 ligand is both necessary and sufficient for regulating the mandibular patterning^37^. Third, HAND2, DLX5 and DLX6, the downstream targets of *Edn1*, are expressed only in the CNC cells of the mandible but not in the maxilla^38^, and finally TWIST1 forms a heterodimer with HAND2 to regulate downstream target genes in mandibular prominences^5, 39^. While we favor the Endothelin1 molecule as an important mediator of this intercellular interaction, we recognize that the treatment of the mandibular cultures with EDN1 partially rescued the mutant phenotype only at the E10.5-E11.5 during mandibular development. However, our data did not address the critical time points when *Edn1* and its cognitive receptor *Ednra* are critical for mandibular patterning at earlier time points than E10.5 according to previous publications^40, 41^. Our partial rescue data did not also exclude the possibility that other signaling pathways might be involved at later time points. Our data showed that EDN1, DLX6 and DLX5 are highly expressed at E12.5 and their expression is relatively reduced in mutant embryos compared to wild type littermates. Our *in vivo* data also showed that the expression of EDN1 was remarkably reduced at protein level in mutant embryos at E10.5 and E12.5. In addition, the *ex-vivo* partial rescue of double heterozygous mutant embryos with EDN1 peptides suggests that the mandibular CNC cells are responsive to EDN1 molecule at E10.5 and E11.5. These results are in agreement with previously published studies that showed that injection of human EDN1 in zebrafish embryos two stages after the migration of CNC cells to pharyngeal arches rescued the Edn1^−/−^ phenotype^42^. Another study used *in vivo* chimera of mouse embryos injected with Ednra mutant embryonic stem cells suggested that Ednra/Edn1 signaling pathway plays a role in post migratory neural crest cell differentiation into bone and cartilage by selecting for Ednra positive cells in these tissues^41^. Moreover, a recent publication using the Wnt1-cre line to overexpress Edn1 in CNC cells suggests that the defects observed in transgenic mice are in the CNC-derived tissues and most likely emerged at the differentiation stage and not only during the early stage of migration and patterning^43^. Collectively, these data suggest that Edn1 may play an important in early stages of CNC cell proliferation and differentiation into cartilage and bone.

A non-cell-autonomous genetic interaction between transcription factors is a mechanism used in development. For example, in sea urchin, knockdown of multiple transcription factors led to altered expression of a different transcription factor in an adjacent cell type^44^. In *Xenopus*, Zic1 transcription factor in the neural plate regulates intercellularly the expression of Six1 and Foxi1c transcription factors in the pre-placodal region through controlling the retinoic acid ligand that is secreted from neural plate into the extracellular matrix of pre-placodal cells^45^. A unique aspect of this study is the presence of a non-cell-autonomous genetic interaction between transcription factors in a mammalian system. Furthermore, the cross between the *Irf6* and *Twist1* generated a synthetic semi-lethal interaction, a genetic phenomenon that was described by Dobzhansky^46^. This interaction is “semi-lethal” because the phenotype of the double heterozygote is lethal, but not completely penetrant. We hypothesize that a cross between transcription factors that share morphogenic functions but are expressed in adjacent cell types is a top-down approach to delineate signaling pathways essential for craniofacial development.

Since the phenotype *Irf6*, *Twist1* double hets is loss of mandibular bone, we expected to observe high levels of cell death or reduction of mesenchymal cell proliferation or both. Previous studies demonstrated that TWIST1 directly interacts with P53 protein to inhibit its pro-apoptotic activity in normal and cancer cells^30, 47^. Our data are consistent with this inhibitory regulation as we detected high levels of P53, BAX and activated-Caspase 3 in mandibular tissues of mutant embryos, while the level of cell proliferation was reduced. The rescue experiment using p53 heterozygous mice to reduce cell apoptosis is consistent with the hypothesis that cell death in the mandibles of affected embryos is p53-dependent as a consequence of reduced expression of Twist1.

In this study, we observed that deficiencies in *Irf6* can lead to mandibular hypoplasia and cleft palate. Since birth defects with small mandible such as Treacher-Collins Syndrome (OMIM #154500) and Pierre Robin sequence (OMIM #261800) are highly associated with cleft palate^48, 49^, we suggest that altered mandibular development may also contribute risk for cleft palate in *TWIST1*- and *IRF6*-related disorders. To support this hypothesis, a recent report identified a likely disease-causing mutation in *IRF6* in a patient with Pierre Robin sequence^50^. Interestingly, identifying the genetic risk factors that cause small mandible might help to discover the missing heritability in isolated orofacial clefting. In addition to agnathia, affected compound heterozygous embryos also had low-set ears and holoprosencephaly. This combination of phenotypes is observed in patients with agnathia-otocephaly complex (AGOTC; OMIM #202650), suggesting a novel animal model for AGOTC. Previous studies found mutations in the *PRRX1* in patients with AGOTC^16, 51–53^. However, mutations have only been found in 15% of patients with AGOTC. Thus, we hypothesize that mutations in the signaling pathway identified here might contribute risk for AGOTC as well. This hypothesis is supported by the recent report of a genetic interaction between *PRRX1* and *TWIST1* to promote cellular invasion during embryogenesis and in cancer cells^54^. Future studies are needed to better understand the association between the function of these transcription factors in craniofacial development and their dysfunction in cancer.

## Methods

### Mice handling and genotyping

Mice generation on C57BL genetic background and genotyping for *Irf6*, *Twist1*, and *Trp53* mutant alleles were performed as previously described^22, 23^. Animal use procedures were approved by the Animal Welfare Committee (AWC-13–055) at the University of Texas Health Science Center at Houston and followed the National Institute of Health guidelines. The desired age of embryos for extraction was determined based on the presence of copulation plug and size of pregnant females’ abdominal area. Pregnant females were euthanized with CO_2_ followed by cervical dislocation. Incision from the lower abdomen to the epigastric region was done for the extraction of embryonic sacks. Individual embryos were isolated and submerged in cold phosphate buffer solution (PBS) for further analysis. A congenic heterozygous mouse strain for *p53* was used to generate triple allelic embryos with *Irf6* and *Twist1*. Affymetrix Mouse Genome 430 Microarray data from Irf6 null dorsal skin at embryonic day 17.5 was analyzed for the expression level of several candidate genes that are known to affect the development of mandible relative to wild type littermate^23^.

### Quantitative gene expression analysis

Total RNA was extracted from mandibular tissues of *Irf6* null and wild type murine embryos at E10.5, E12.5 and E14.5 to measure expression level of several genes. RTqPCR experiments were performed as previously described^55^. Primers for *Twist1*, *Runx2, Grhl3* and *Gapdh* were described previously^56, 57^. *Gapdh* expression level was used in each plate for normalization purposes. Four technical replicates of each treatment were used to calculate the average of relative gene expression and the bar represents the standard deviation. Immunoblot was performed in mandibular tissues for the wild type and mutant embryos to detect the total protein levels at E10.5 and E12.5 time points as previously described^29^. Odyssey Li-Cor system was used to visualize the protein bands and GAPDH was used as a loading control. We performed multi-variants ANOVA test for the normalized expression data to determine the significant difference among the mean values. A p-value less than 0.05 is considered statistically significant.

### siRNA transfection

HEK293 cells were cultured in DMEM medium until 80% confluence and then transfected with 200 nM of validated siRNAs for human TWIST1 (Invitrogen, CA) and corresponding control scrambled siRNAs using lipofectamine reagents. Twenty-four hours after transfection, cells were harvested and total RNA was extracted for RT-PCR. Similarly, the effect of reducing *TWIST1* expression on *IRF6* enhancer activity was tested in luciferase assay using the same concentration of TWIST1 siRNAs and the resulted were compared to enhancer activity alone and to luciferase construct without enhancer element. Five replicates of each treatment were analyzed 24 hours after transfection by real-time RT-PCR.

### ChIP-qPCR

To test the direct binding of TWIST1 protein to IRF6 enhancer element, we performed chromatin immunoprecipitation (ChIP) followed by quantitative real time PCR in human keratinocytes (HaCaT) cells. The binding of PolII at the promoter region of IRF6-transcription start site (TSS) was used to detect the transcriptional state of *IRF6* gene. The PCNA promoter was used as an internal control for the efficiency of chromatin immunoprecipitation and specificity of TWIST1 and PolII antibodies. To quantify the amount of immunoprecipitated DNA that was pulled down by TWIST1 antibodies, we used SYBR Green Kit (Applied Biosystems, Foster City, CA) to measure relative DNA fraction of the total input by a real time PCR. We included five replicates of each treatment, applied the standard curve method for quantifications, and normalized the data to the level of immunoprecipitated DNA that was pulled down by H3 antibodies as previously described (Fakhouri *et al*. 2014). Immunoprecipitated DNA using IgG antibodies were served as negative control for background signal and non-specific binding.

### Mandibular imaging and measurements

We used NIS Elements AR software on a Nikon’s stereomicroscope to image skeletal preps. We then mapped morphometric landmarks to measure the length, width and area of the mandible (Nikon SMZ800). For the length, two measurements were taken in order to account for the curved structure of the mandible. The first measurement was taken from the posterior point of the condylar process (point 1) to the inferior point of the mandibular process (point 2). The second measurement was taken from point 2 to the most anterior point of the mandible excluding the incisor bone primordium (point 3). These two measurements were added together to give the total length. We calculated the average using two measurements from two distinct landmarks. The first measurement was taken at the widest section of the mandible, from the molar alveolus of dentary (point 4) to point 2. The second measurement was taken at the narrowest section of the mandible, from point 3 to the anterior-superior point of the mandible (point 5). Lastly, the area of the mandible was measured using the area tool (Supplementary Fig. S3). We performed multi-variants ANOVA test for the quantitative measurements of the mandible for the different genotypes to determine the significant difference among the mean values.

### Immunofluorescent (IF) Staining

Expression of transcription factors, ligand and pro-apoptotic proteins were analyzed on paraffin sections from heads of wild type and double heterozygous mutant embryos at different embryonic times points. Bromodeoxyuridine (BrdU) is a thymidine analog that is incorporated into DNA during the S phase of the cell cycle to mark proliferating cells. Caspases, or cysteine-aspartic proteases, are a family of cysteine proteases that play essential roles in apoptosis. We used activated-Caspase 3 as a marker to detect the cells that are going into apoptosis. Immunostaining was performed as previously described^58^. Briefly, murine tissues were deparaffinized and rehydrated in a series of ethanol dilutions. Slides were boiled for 10 minutes in 10 mM sodium citrate buffer for antigen retrieval. Sections were blocked with 10% normal goat serum in 1% PBS-BSA for 1 hour, then incubated overnight at 4 °C with the following primary antibodies: monoclonal mouse anti-Twist1 (1:150, Abcam, MA) and polyclonal rabbit anti-Irf6 (1:500)^58^, rabbit anti-Edn1, rabbit anti-casp3, rabbit anti-p53 (1:200, Abcam, MA), rabbit anti-Bax (1:200, Santa Cruz), BrdU (1:250, Abcam, MA). The secondary antibodies were goat anti-rabbit (A21429, Molecular Probes, CA) and goat anti-mouse (A11029, Molecular Probes, CA). We marked nuclei with DAPI (D3571, Invitrogen, CA). An X-Cite Series 120Q laser and a CoolSnap HQ2 photometric camera (Andor Neo/Zyla) installed in fluorescent microscope (Nikon Eclipse Ni) were used to capture images from a Plan APO 40x/0.95 DIX M/N2 objective.

### Immunohistochemical (IHC) Assay

IHC was also used for localization of biomarkers and to detect differentially expressed proteins when autofluorescence and light scattering interfered with image quality. IHC was used for DLX5, DLX6, HAND2 and P53. For IHC, sections were deparaffinized and rehydrated in a series of ethanol dilutions. The sections were boiled for 30 seconds in 10% blocking solution (Biocare, MX, DV-200), washed with 0.05% PBS-Triton, then incubated with the following primary antibodies: rabbit polyclonal anti-DLX5 (Abcam, MA, YH05211C), polyclonal goat anti-DLX6 (Santa Cruz, CA, sc-18154), monoclonal mouse anti-HAND2 (Santa Cruz, CA, sc-130629), monoclonal mouse anti-P53 (Abcam, MA, GR1277-1), diluted in horse serum overnight at 4 °C. Next day, they were washed with 0.05% PBS-Triton and then incubated with the secondary anti-mouse IgG, anti-rabbit IgG, or anti-goat IgG (Vector lab PK-7800, CA) for 20 minutes at room temperature and then washed with 0.05% PBS-Triton. We then treated the samples with tertiary antibody streptavidin-peroxidase complex (Vector lab SK-4800, CA) for 5 minutes at room temperature. Finally, the sections were treated with chromagen (Impact Novared Kit SK-4805) followed with hematoxylin (Fisher scientific, 123022) for nuclear staining. The hematoxylin was rinsed off with distilled water and the slides mounted with cytoseal (Fisher Scientific, PA).

### Skeletal preparation

To determine the effect on bone and cartilage, skeletal preparation was performed on wild type and mutant murine pups. Internal organs were removed from pups through a small incision in the abdominal area and then fixed in 95% ethanol for 1–2 days. After replacing the ethanol with 2% KOH until the embryo’s skin becomes transparent, we incubated the samples in acetone for 5 hours and stained them with Alcian blue solution overnight. Next day, the samples were washed with a series of descending ethanol concentrations and then replaced with Alizarin red solution for 6 hours. After that, embryos or pups were submerged in 20% glycerol and 1% KOH to remove excessive stain. All steps involving solutions were done under room temperature with gentle agitation.

### *Ex-vivo* mandibular explant

To test our model, we attempted to rescue mandibular hypoplasia in double heterozygous embryos by treating mandibular organ explants with Endothelin1. With this system, we were able to manipulate the concentration, time point and method of Endothelin1 application. Mandibles were dissected from E10.5-E11.5 wild type, hets and mutant embryos and grown at 37 °C using a culture system containing BGJb medium (Gibco BRL, NY) with L-glutamine, 140 μg/ml of ascorbic acid (S25184, Fisher Scientific, PA) and 100 U/ml of streptomycin-penicillin (SLBB9308, Sigma Co). Non-essential and essential amino acids were added for proper growth and development. Media was changed every day for the first 2 days, than every two days for the rest of the incubation period. Because proper gas transfer and exchange are important for the growth of the mandible, the media barely submerges the mandible in the culture dish. For the rescue experiments, 2 mm-diameter agarose beads saturated with a solution of 0.1 μg/ml of Endothelin1 peptide (89143–252, Enzo Life Sciences) were placed on each side of the mandibles. After 66 hours of incubation, the explants were carefully removed and skeletal staining was performed to detect changes in cartilage and bone formation.

## Electronic supplementary material

Supplementary Information
