# Supplementary material for: Intercellular Genetic Interaction Between Irf6 and Twist1 during Craniofacial Development
Source: Sci Rep. 2017 Aug 2;7:7129. doi: 10.1038/s41598-017-06310-z (PMC5540929; doi:10.1038/s41598-017-06310-z)
Supplement: Supplementary file 1 — Supplementary Information [file 41598_2017_6310_MOESM1_ESM.doc]

**Intercellular Genetic Interaction Between *Irf6* and *Twist1* during Craniofacial Development**

Walid D. Fakhouri^1,2,3^*, Kareem Metwalli^1^, Ali Naji^1^, Sarah Bakheit^1^, Angela Quispe-Salcedo^1^, Larissa Nitschke^4^, Youssef A. Kousa^5^, Brian C. Schutte^4,5,6^

**
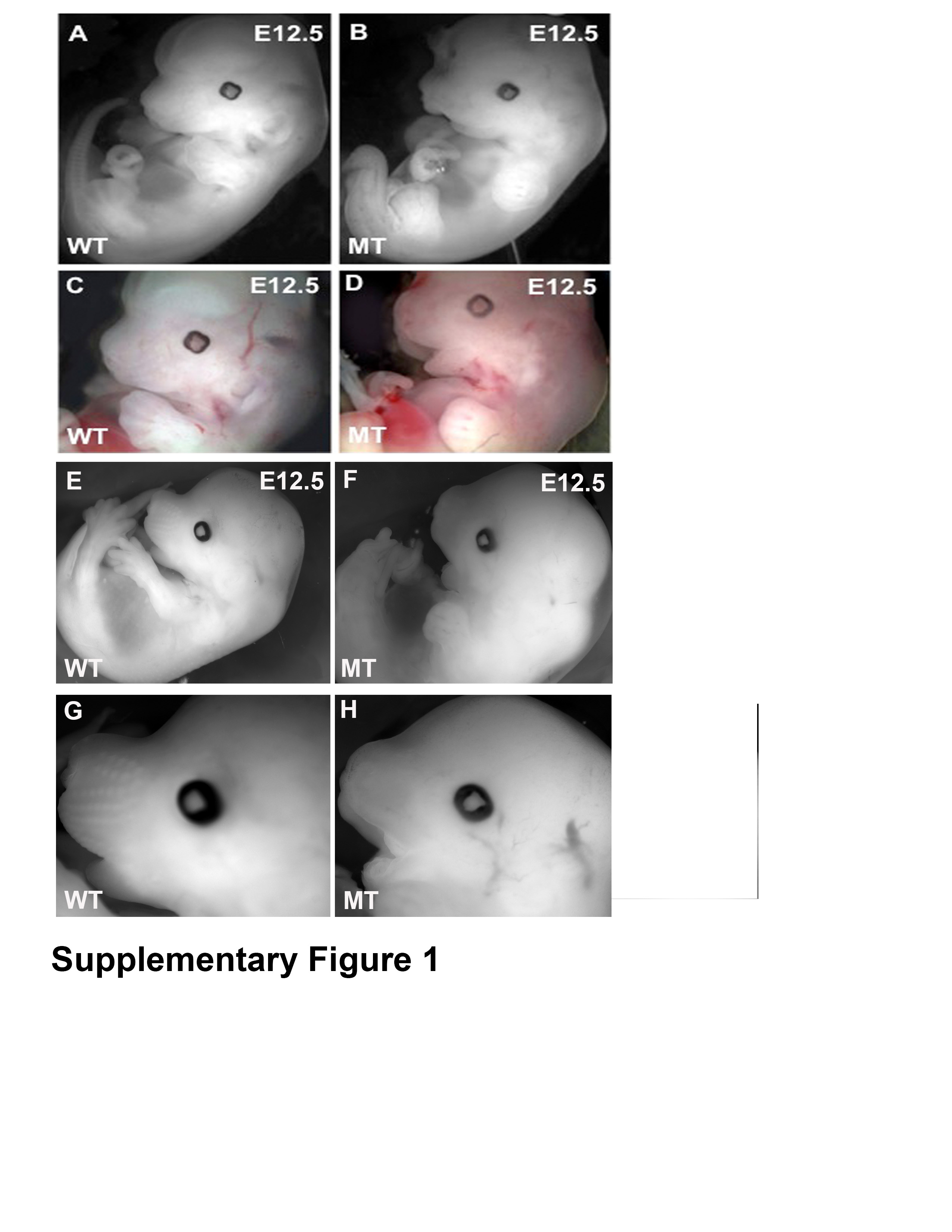
**

**Supplementary Figure 1**. **Stereomicroscope images of murine embryos at E12.5**. When compared to wild type (A, C, E, G), double heterozygous mutant embryos show undersized mandible, limbs and tail (B, D).


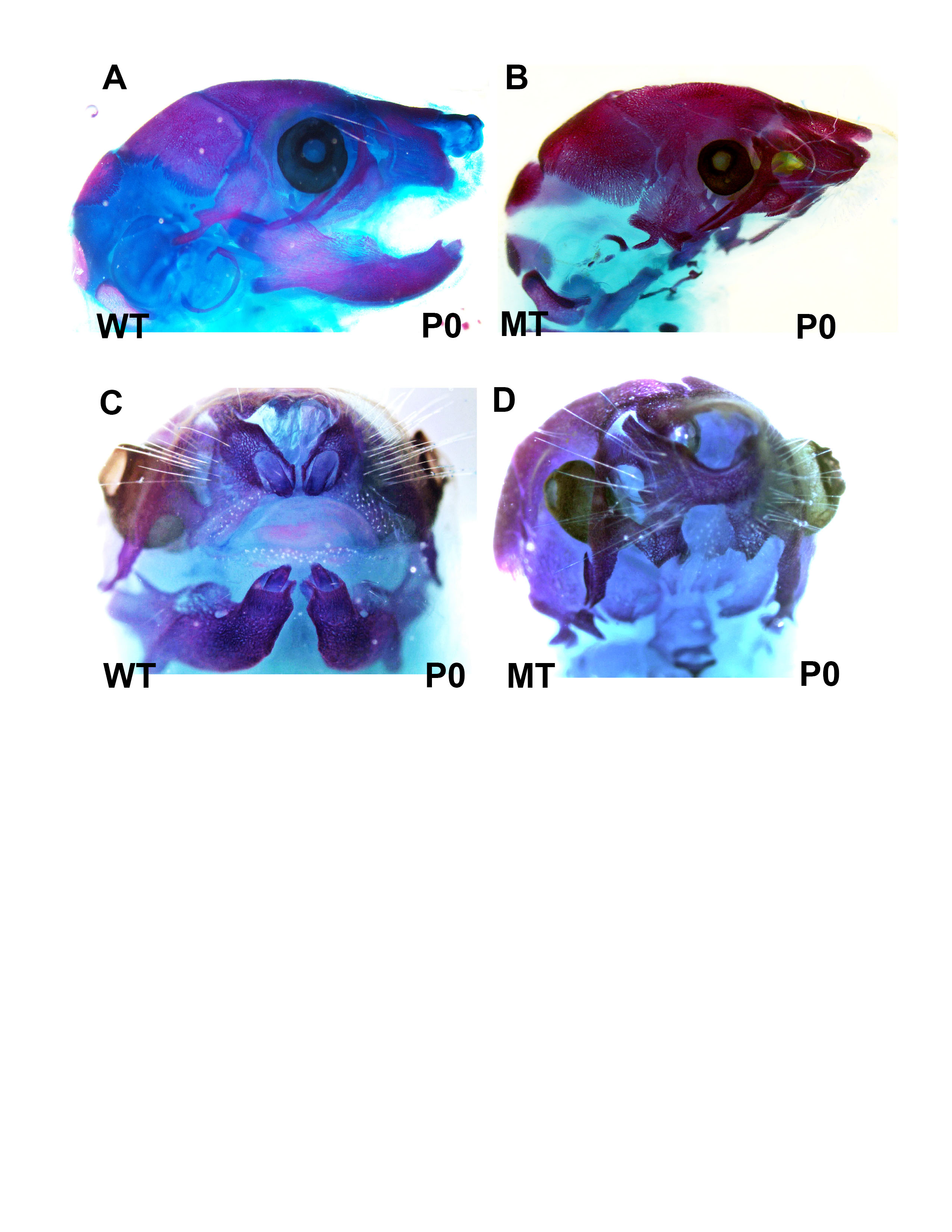


**Supplementary Figure 2. Craniofacial skeleton of murine embryos at birth**. Representative images of newborn wild type pups (A, C) and double heterozygous mutant pups (B, D). Side view of wild type (A) compared to mutant pups (B) shows lack of mandible (B) without other abnormalities in skull and frontal nose. Front view of wild type (C) compared to mutant (D) shows lack of mandible and fusion of maxillary bone at the facial midline (D).


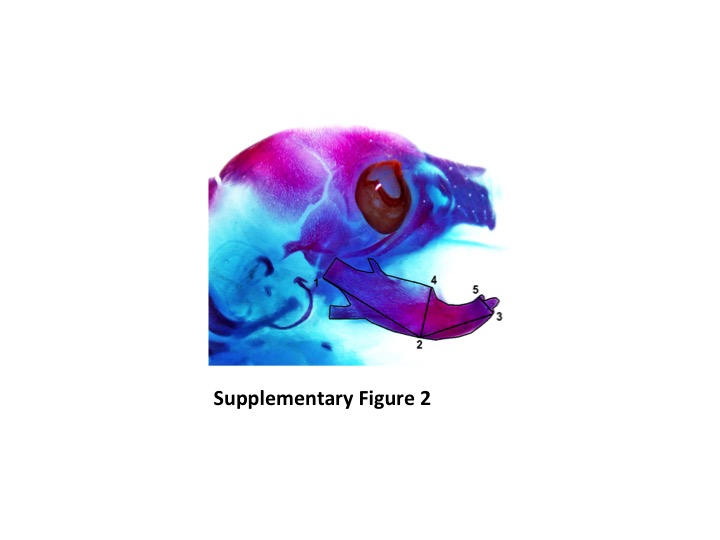


**Supplementary Figure 3**. **Image of wild type (*Irf6*+/+, *Twist1*+/+) murine embryo at E17.5**. The image is an illustrative method of taking mandibular measurements. Numbers represent points at which measurements of length and width of mandible were taken. The length is the sum of the line from point 1 to point 2 and the line from point 2 to point 3. The width was the average of the line from point 4 to point 2 and the line from point 5 to point 3. Area was measured by outlining the mandible. Definitions of landmarks: 1. posterior point of the condylar process; 2. inferior point of the mandibular process; 3. most anterior point of the mandible; 4. molar alveolus of dentary; 5. anterior-superior point of the mandible.


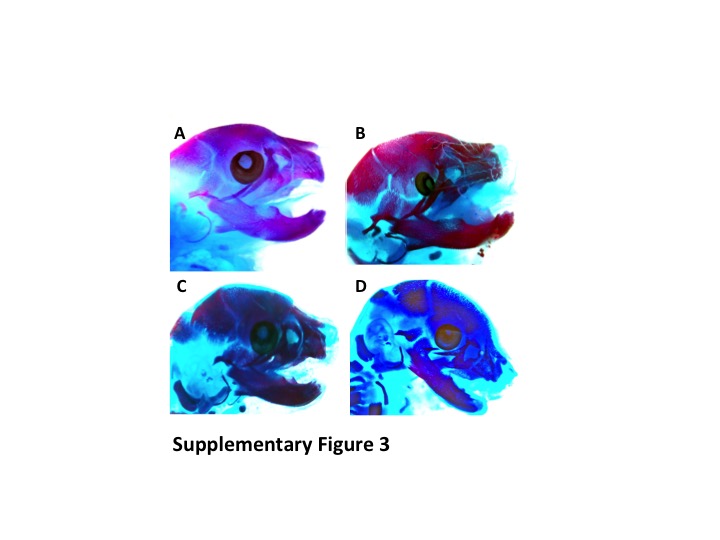


**Supplementary Figure 4. Craniofacial skeleton of murine embryos**. Representative images of four embryos of different genotypes showing craniofacial skeleton used for mandibular measurements. Wild type (A), *Irf6* single heterozygous (B), *Twist1* single heterozygous (C) and double heterozygous for *Irf6* and *Twist1* (D).


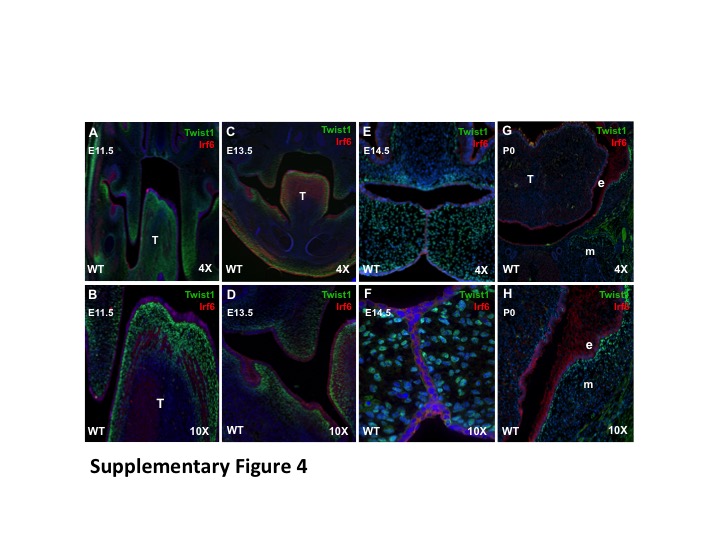
**Supplementary Figure 5**. **Immunofluorescent images of *Irf6* and *Twist1* expression at different embryonic time points**. *Irf6* is predominately expressed in oral epithelium (red), while *Twist1* is expressed in the adjacent mesenchymal cells (in green) at E11.5 (A, B) and thereafter (C-F). The expression pattern of *Irf6* and *Twist1* continues in the same tissues postnatally (P0) (G and H).


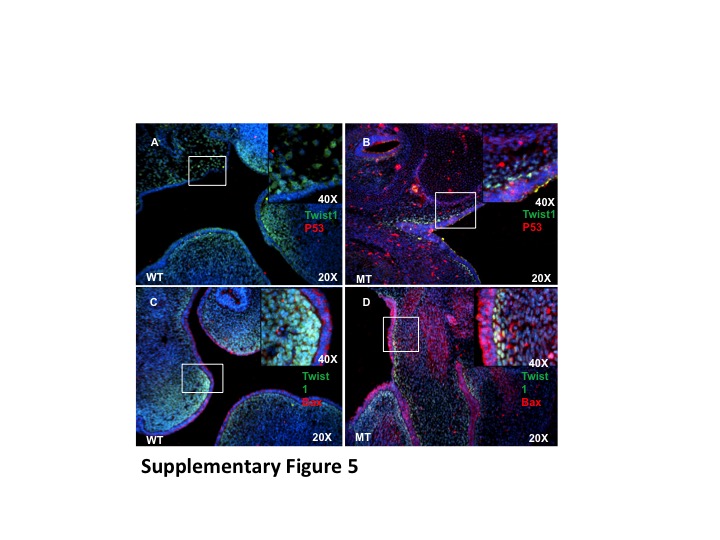


**Supplementary Figure 6. Dual immunofluorescent staining for the expression of TWIST1 with P53 and TWIST1 with BAX**. Relative to the wild type (A and B), TWIST1 expression is reduced in mesenchymal cells while P53 and BAX are ubiquitously increased in double heterozygous affected embryos (B and D). Coronal sections of embryonic heads were used to determine the expression level at E12.5.


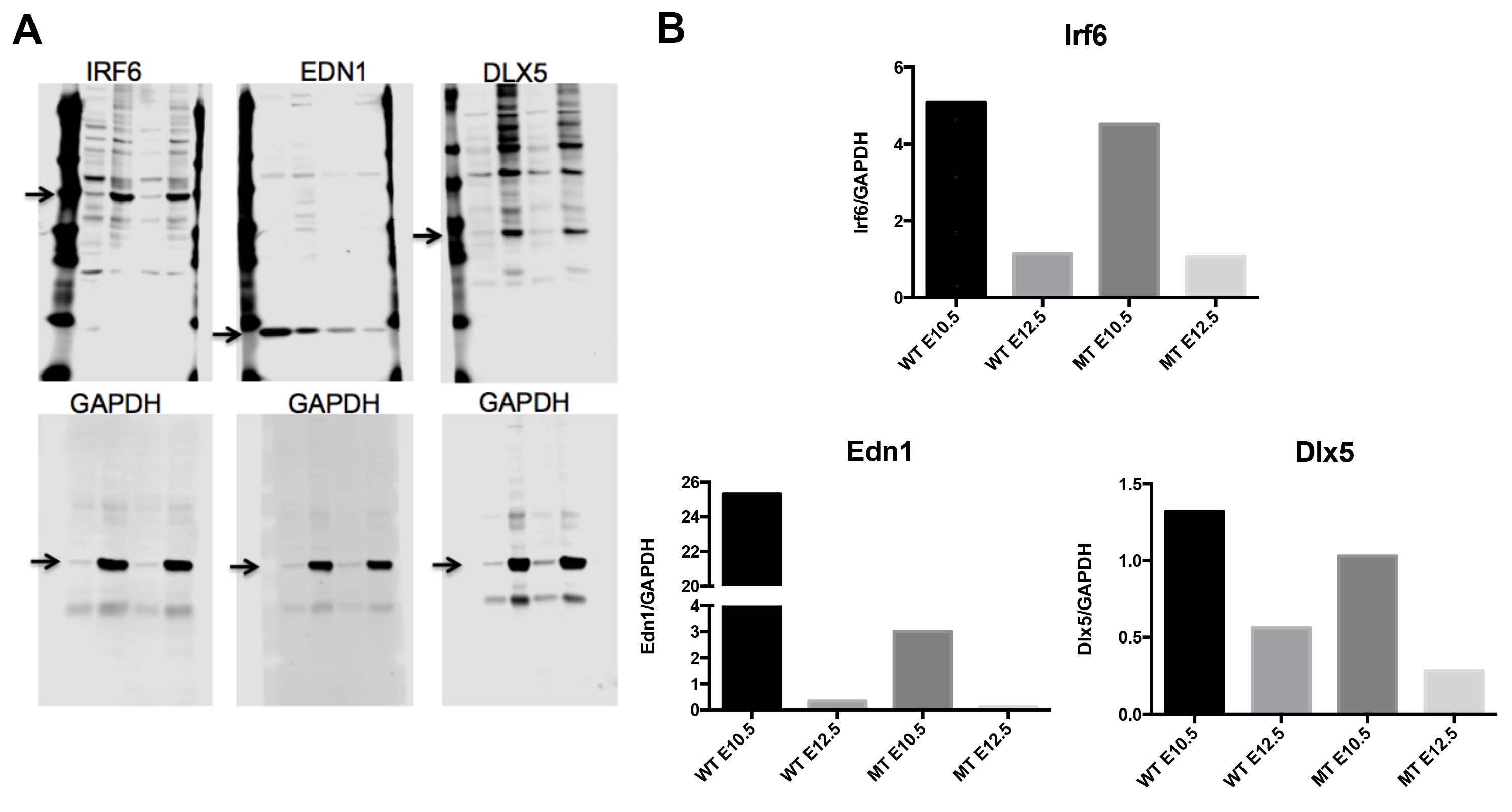


**Supplementary Figure 7**. **Full-length immunoblots and** **quantification of bands for genes expressed in mandibular tissues of mouse embryos**. Blots of IRF6, DLX5 and EDN1 show bands with the accurate protein size, however other multiple bands were observed in IRF6 and DLX5 blots (A). Normalized protein level of IRF6, EDN1 and DLX5 is more expressed in wild type tissues at E10.5 and to a lesser extend at E12.5 (B). In mutant embryos, expression of Irf6 is slightly reduced at E10.5 but no change at E12.5 (B). However, EDN1 protein amount is remarkably reduced at both time points, while DLX5 amount is reduced more at E12.5 (B).


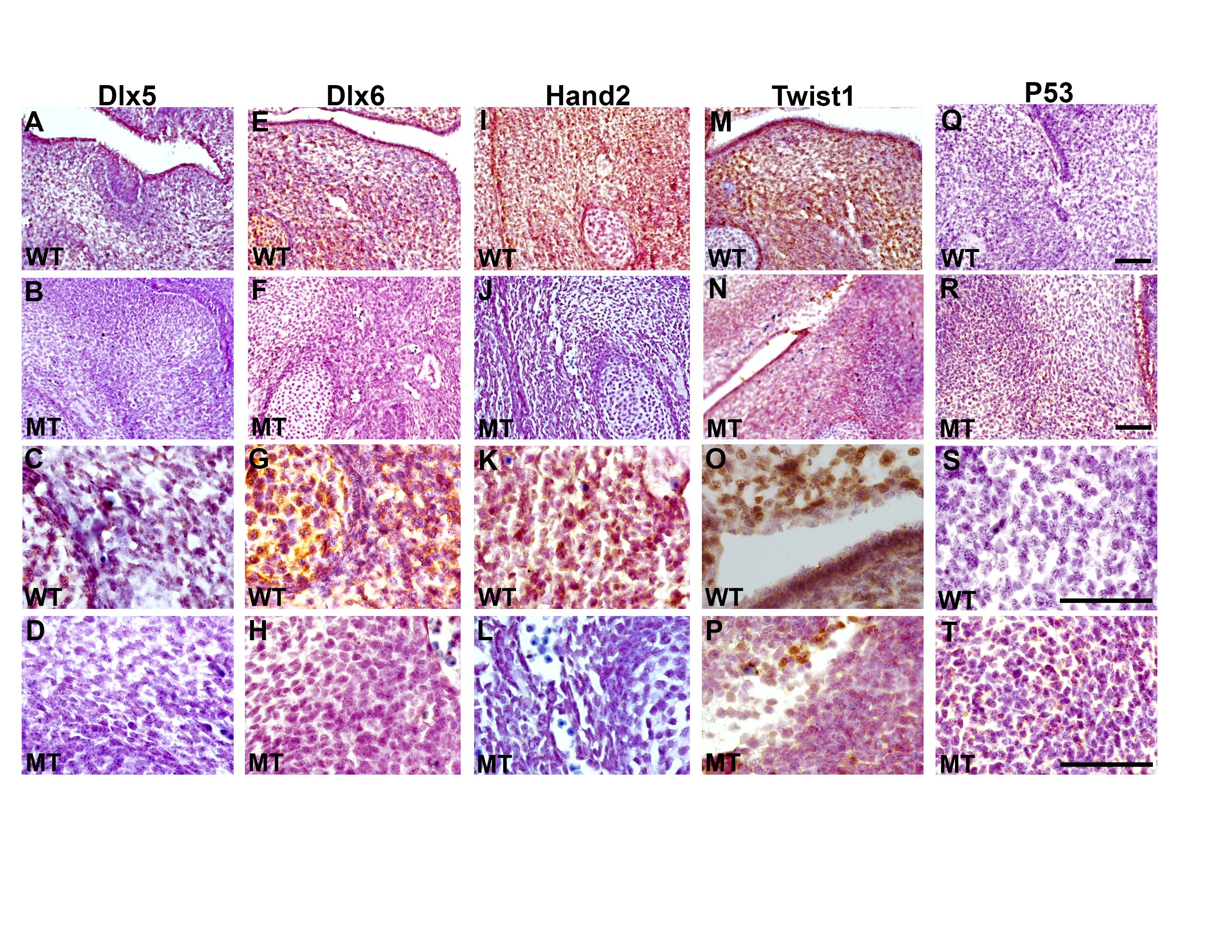


**Supplementary Figure 8**. **Protein expression of DLX5, DLX6, HAND2, TWIST1 and P53 in mandibular tissues**. The counter staining color used in the immunohistochemistry is purple for nuclei and protein expression is in red. When compared to the wild type samples (top row and third raw), expression intensity of DLX5 (A, C), DLX6 (E, G), HAND2 (I, K) and TWIST1 (M, O) reduced in double heterozygous mutant embryos for DLX5 (B, D), DLX6 (F, H), HAND2 (J, L) and TWIST1 (N, P) in mandibular processes at E12.5. In contrary, expression of P53 is low in wild type (Q, S), but increased in mutant (R, T) at E12.5. Scale bars = 20 μm.

**
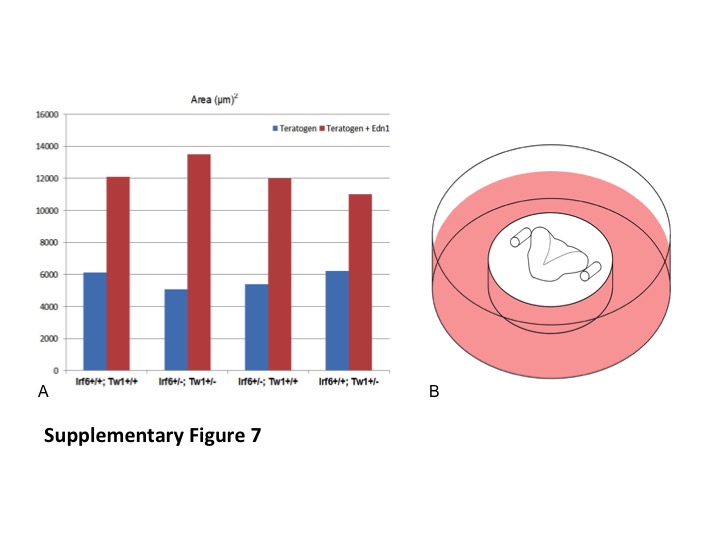
**

**Supplementary Figure 9. Area measurements of Meckel’s cartilage and illustration of the organ culture plate.** Exogenous treatment with Edn1 peptides remarkably increases the area of Meckel’s cartilage of mandibular organs in all different genotypes (A). The setup of the mandibular organ and the agarose cylinders used to deliver the exogenous Edn1 peptides is illustrated in the schematic drawing (B).
